# The impact of the COVID-19 pandemic on the provision of HIV/AIDS-related services in Iran: a qualitative study

**DOI:** 10.1186/s12913-023-09407-6

**Published:** 2023-05-03

**Authors:** Zahra Jaafari, Hossein Mirzaei, Yousef Moradi, Naser Nasiri, Soheil Mehmandoost, Mehrdad Khezri, Fatemeh Tavakoli, Samaneh Abbaszadeh, Hamid Sharifi

**Affiliations:** 1 Student Research Committee, Shiraz University of Medical Sciences, Shiraz, Iran; 2 HIV/STI Surveillance Research Center, WHO Collaborating Center for HIV Surveillance, Institute for Futures Studies in Health, Kerman University of Medical Sciences, Kerman, Iran; 3 Department of Epidemiology and Biostatistics, Faculty of Medicine, Kurdistan University of Medical Sciences, Sanandaj, Iran; 4 Social Determinants of Health Research Center, Research Institute for Health Development, Kurdistan University of Medical Sciences, Sanandaj, Iran; 5 Department of Epidemiology, New York University School of Global Public Health, New York, NY USA

**Keywords:** Health Services accessibility, HIV/AIDS, COVID-19, Qualitative research, Iran

## Abstract

**Background:**

Providing services to people living with HIV (PLWH) faced many challenges during the COVID-19 pandemic. This study aimed to examine the impact of the COVID-19 pandemic on providing HIV/AIDS-related services in Iran.

**Methods:**

In this qualitative study, the participants were included by purposive sampling between November 2021 and February 2022. Virtually focused group discussion (FGD) meetings were conducted with the first group including policymakers, service providers, and researchers (n = 17), and the interviews were conducted telephonic and face-to-face using a semi-structured guide with the second group including people who received services (n = 38). Data were analyzed by content analysis using the inductive method in MAXQDA 10 software.

**Results:**

Six categories were obtained, including mostly affected services, ways of the effect of COVID-19, healthcare systems reaction, effects on social inequality, opportunities created by the pandemic, and suggestions for the future. In addition, people who received services believed the COVID-19 pandemic has affected their life in several ways, including getting COVID-19, mental and emotional problems during the pandemic, financial problems, changes in the care plan, and changes in high-risk behaviors.

**Conclusion:**

Considering the level of community involvement with the issue of COVID-19 and the shock caused by the pandemic, as mentioned by the world health organization, it is necessary to improve health systems’ resilience for better preparedness for similar conditions.

## Background

According to the Joint United Nations Programme on HIV/AIDS (UNAIDS) report in 2021, around 38 million people were living with HIV (PLWH) worldwide, and 650,000 died due to this disease in this year [1]. The number of PLWH in Iran is also reported to be 53,000, of which 3,500 have died in 2021 [2]. These people may be exposed to acute respiratory infections such as COVID-19. To limit the transmission of the virus in communities, various care measures such as lockdowns, social distancing, the use of masks and gloves, and the use of disinfectants have been recommended. But most importantly, staying at home and not going out for an indefinite period is one of the most important and influential measures to control the COVID-19 pandemic [3, 4]. According to the studies report, PLWH experience more adverse health outcomes (for example, increased anxiety and depression and decreased sleep quality) than the general population [5, 6]. In addition, PLWH receiving antiretroviral therapy (ART) have a better prognosis for COVID-19 than those not receiving treatment [7].

Illness and death caused by the COVID-19 pandemic, closure of service provider centers or reduced access to services, observance of social distance or other measures and care to deal with the spread of the SARS-CoV-2 virus are among the factors that prevent PLWH and those at higher risk for HIV and need harm reduction services from accessing medical services and diagnosis [8, 9]. In addition to the risk of COVID-19 disease, indirect effects such as unemployment, depression, and widespread social anxiety, which can play a synergistic role as important aggravating factors in the occurrence of other health outcomes, are among the other consequences of COVID-19 in PLWH [10, 11]. There are also concerns about the financial and human resources allocated to reduce the effects of the COVID-19 pandemic in developed and developing countries, that the allocation of these resources will prevent or reduce the allocation of resources to HIV prevention programs [12, 13].

Public access to HIV care and treatment is an essential global priority and has been emphasized as a global commitment in the United Nations General Assembly meetings [14]. By suppressing HIV in the blood, ART plays a vital role in preventing the transmission of the virus and improving the lives of patients [15]. ART is effective in increasing the life expectancy of PLWH and reducing co-infections. In addition, late referral of patients to be treated, while increasing the possibility of transmitting the disease to others and more deaths, will also increase the cost of treatment [16, 17]. In a study in low-income and middle-income countries, it was estimated that the greatest impact on HIV is from interruption to antiretroviral therapy, which can occur during the COVID-19 pandemic with high demands on the health system [18]. Also, access to counseling, testing, and other services can prevent late diagnosis and reduce the possibility of transmitting the virus to others, preventing many complications and deaths [16]. During the COVID-19 pandemic, a study in Guatemala showed that deaths from opportunistic infections increased by 10.7% in 2020 compared to 2019. Also, the clinical samples sent for the diagnosis of opportunistic infections decreased by 43.7% in 2020. Therefore, it is essential to ensure easy access to services for PLWH [16].

Currently, there is little and contradictory data and information in the world, especially in Iran, regarding the impact of the COVID-19 pandemic on the provision of services related to HIV/AIDS. This lack of evidence and its contradictions have prevented the creation of international and national programs to provide care to PLWH and support these people during the COVID-19 pandemic. In Iran, quarantin and travel bans have been implemented several times. On the other hand, in administrative and government centers (related to health, etc.), services were provided in absentia for almost a long time. Investigating the effects of the COVID-19 pandemic and of interventions to control the pandemic by providing HIV-related services and care is crucial for better management. Considering that qualitative research is a method to achieve a deeper understanding of a phenomenon, it also shows the broad patterns behind the phenomenon that is being researched; therefore, this study aimed to assess the impact of the COVID-19 pandemic on the condition of HIV/AIDS-related services in Iran.

## Methods

### Study population and setting

This qualitative study was conducted in three provinces of Iran, including Tehran (the capital), Khorasan Razavi (north), and Kerman (south). Between November 2021 and February 2022, two groups were interviewed for this study. The first (group A, n = 17) was the policymakers, service providers, and researchers in the field of HIV. The participants in this group were recruited from interested organizations, including the Ministry of Health and Medical Education and the welfare organization, the two leading HIV-related services providers in the country. Service providers were recruited from different levels, including the highest level of planning and decision-making regarding HIV-related programs and the level of direct providers of services to individuals. The inclusion criteria for this group included former and current managers and officials of the AIDS Department of the Ministry of Health and the Harm Reduction Office of the Welfare Organization. Also, we included provincial officials or experts on the HIV program, physicians, or counselors working in voluntary counseling and testing centers (VCT) in the interviews.

The second (group B, n = 38) was the group of people who received services, which included PLWH (n = 26) and also people who were at higher risk of HIV (n = 12). These people were referents to the VCT or non-governmental organizations (NGOs). The VCT is a referral center for providing care for PLWH; the main clients of the VCT included the following people: PLWH, people at higher risk (e.g., people who inject drugs, female sex workers, men who have sex with men), and couples who plan to get tested for HIV before marriage [19]. In these centers, various services are offered to people for free, including conducting confidential HIV tests, counseling before and after the test, and health promotion interventions to prevent HIV transmission (for example, harm reduction services such as condoms and methadone). They were also treating patients and providing psychological and emotional services to them.

### Procedures

The protocol of this project has been reviewed and approved by the Ethics Committee of the National Institute for Medical Research Development of Iran (Code of ethics: IR. NIMAD. REC.1400. 107). All procedures were performed in accordance with relevant guidelines. The study was designed and conducted by an experienced HIV research team. Participants were selected based on purposive sampling. Informed consent was obtained from all subjects. To make the necessary coordination, group A was contacted, and arrangements were made to hold focused group meetings with the experts of these organizations. Because the discussion sessions were held during the COVID-19 pandemic, restrictions on travel and preventing gatherings were held virtually and through Skyroom. Four focus group discussion (FGD) meetings were conducted with an interviewer to guide the discussion and create interaction between the participants and a coordinator to take notes and record the voice of the participants. Each FGD continued until information saturation was reached, so no new information was found as the discussion continued. The approximate time of each FGD lasted from 60 to 95 min.

In group B, semi-structured interviews were conducted with 38 participants to collect information. The interviews were conducted in each city by an interviewer from the same city to create the same conditions in all the interviews. The interviewers were given the necessary training, and the same questions and interview guide were prepared and provided. After selecting each person, 750,000 IRRs (around 2.5 USD) were paid to Khorasan Razavi and Kerman participants and 1,500,000 IRRs (about 5 USD) in Tehran for time and travel expenses. The interviews were conducted using face-to-face interviews and a semi-structured guide. The approximate time for the interview was between 15 and 30 min.

Interviewers were conducted in a private room in a VCT or NGO in each city. If the interviewees were willing to participate in the study, they were referred to the interviewers by VCT or NGO officials. Interviewers began the interview by introducing themselves, stating the study’s objectives, and asking participants to talk about their experiences of accessing HIV services and care since the beginning of the COVID-19 pandemic. The interview guide was prepared by reviewing the literature. Three experts and researchers in HIV care and clinical services evaluated and modified the questions if necessary.

In addition to the questions in the interview guide, the researcher could also add questions during the interview. Some of the questions included for group A the following: “Has there been a change in the financial resources and budget allocated to HIV prevention, treatment, and research services during the COVID-19 pandemic?”, “In your opinion, during the COVID-19 pandemic, HIV prevention services and access to them for people at risk and infected in the country have faced challenges and problems?”, “In your opinion, should PLWH be prioritized to receive services related to COVID-19, especially vaccines?”, “Has any action been taken so far in the country?”; and for group B: “Compared to before the COVID-19 pandemic, has there been a change in your life conditions (changes in your work and income, changes in recreation, risky behaviors, use of preventive measures, social stigma and other people’s behavior towards you, etc.)? “How have these changes affected your disease/infection status?” “During the COVID-19 pandemic, have you ever needed HIV prevention equipment or other harm reduction services but not been able to get them? What was the reason for not receiving the service?”.

### Data analysis

The data were analyzed using the inductive content analysis approach [20]. First, the interviewers transcribed the recorded interviews verbatim and anonymously on the day of the interview. later, codes were extracted based on textual data, and patterns related to participants’ interviews. Then subcategories were found using combining common codes and concepts. After that, categories were formed by combining the subcategories. The research team held weekly meetings to review the codes and subcategories and ambiguities were resolved. During the work process, we collected new information to identify distinct issues. Sampling continued until data saturation was reached and no new concepts were identified during analysis. Additionally, after the researchers achieved data saturation, three more respondents in each group were interviewed to ensure data saturation. The written interviews were read several times by the first and corresponding authors, and if there was any ambiguity, a group discussion was held with all the authors and the ambiguities were resolved. MAXQDA10 software was used to manage and analyze the collected data.

### Trustworthiness

To ensure trustworthiness, four criteria were used: credibility, transferability, dependability, and conformability [21]. For the credibility of the data, attention was paid to confirming the interpretations obtained from the interviews. To investigate the transfer criterion, it tried to describe the characteristics of the participants in detail while using maximum diversity in sampling. The procedures were described in detail to the team members and external observers to ensure the dependability of the data. Discussion in the research team also helped to confirm the findings.

## Results

Most (n = 10, 58.8%) of the participants in group A had PhD degree. The most common fields of study among all of the participants were epidemiology (n = 4, 23.5%), and psychology (n = 4, 23.5%). All of the participants were experienced in the field of HIV or harm reduction services. Also, in group B, there were equal numbers of men and women, most of whom were 40–50 years (65.3%), and most were single (42.3%). In addition, in the group of people at risk of HIV, most of them were men (66.7%), age group less than 30 (33.3%) and 30–40 years (33.3%), and single (58.8%) (Table 1).

**Table 1:** Demographic characteristics of policymakers, service providers, and researchers (N = 17) (Group A), people living with HIV (N = 26), and people at risk of HIV (N = 12) (Group B)

Group A: policymakers, service providers, and researchers			
Variable			Number (%)
Gender	Female		8 (47.1)
	Male		9 (52.9)
Age (Years)	30–40		7 (41.1)
	40–50		8 (47.1)
	> 50		2 (11.8)
Degree of education	MD, or PhD		10 (58.8)
	MSc		2 (11.8)
	BSc		5 (29.4)
Field of study	Epidemiology		4 (23.5)
	Infectious Disease Specialist		3 (17.6)
	Medicine Doctor		2 (11.8)
	Social Studies Specialist		2 (11.8)
	Psychology		4 (23.5)
	Public Health		2 (11.8)
Work experience in the field of HIV	1–5 years		2 (11.8)
	5–10 years		6 (35.3)
	10–15 years		7 (41.1)
	More than 20 years		2 (11.8)
**Group B**			
**Variable**		**People living with HIV (%)**	**People at higher risk of HIV (%)**
Gender	Male	13 (50.0)	8 (66.7)
	Female	13 (50.0)	4 (33.3)
Age (Year)	< 30	0	4 (33.3)
	30–40	3 (11.5)	4 (33.3)
	41–50	17 (65.3)	3 (25.0)
	51–60	6 (23.1)	1 (8.3)
Marital status	Married	6 (23.1)	2 (16.7)
	Single	11 (42.3)	7 (58.3)
	Separated/Widowed	9 (34.6)	3 (25.0)
Education Status	Primary school and low	9 (34.6)	2 (16.7)
	Middle school	12 (46.2)	4 (33.3)
	High school and higher	5 (19.2)	6 (50.0)
Job	Unemployed/ unstable job	20 (76.9)	10 (83.3)
	Employed/stable job	6 (23.1)	2 (16.7)

The group discussion results were classified into six categories. These include the following: (1) mostly affected services, (2) ways of the effect of COVID-19, (3) healthcare systems reaction, (4) effects on social inequality, (5) opportunities created by the pandemic, and (6) suggestions for the future. In addition, the category of ways of the effect of COVID-19 was classified into five subcategories from the perspective of people who received services (Table 2).

**Table 2:** Categories and Subcategories of the impact of COVID-19 on the provision of HIV/AIDS-related services in Iran

Categories	Subcategories
Mostly affected services	Reducing visits to centers for diagnostic tests
	Reducing the access of service providers to patients and people at risk
Ways of the effect of COVID-19	Increasing high-risk behaviors of HIV in society
	Delay in detecting PLWH
	Increasing alcohol and drug use ceremonies instead of other entertainment
	Dedicating the media to COVID-19
	Allocation of resources to the COVID-19 department and lack of resources in the HIV department
	Getting infected with COVID-19 and having unfavorable physical conditions and worsening the condition of HIV
	Losing relationships with peer groups and friends
	Loss of work and income of PLWH
	Increasing the duration of drug storage for people
	Providing door-to-door services by peer groups
	Changing the consumption pattern to injections
	Changing the pattern of drug use from group use to solitary use
	Reducing high-risk sexual relations in the era of COVID-19
Healthcare systems reaction	Providing door-to-door services
	Increasing the distance of people’s visits to receive harm reduction equipment
	Increasing the distance between people’s visits to receive medicine and counseling
Effects on social inequality	Inequality in vaccine prioritization
	Greater impact of COVID-19 on poor and vulnerable people
Opportunities created by the pandemic	Paying more attention to providing online services
	Supplying HIV self-testing services
Suggestions for the future	Pay attention to self-test
	Training employees about providing services in critical situations in the future

**1) Mostly affected services.** Most participants believed that the provision of HIV services had been greatly affected by the COVID-19 pandemic. According to his opinions, these effects were very high at the beginning of the pandemic, and gradually the effects of the pandemic decreased. It can be said that it has returned to normal now. Also, according to the participants’ comments, diagnostic and preventive services are more affected, while treatment services have been affected by the COVID-19 pandemic to a minimal extent.

In this regard, one of the participants said, *“All parts of our organization, including harm reduction, have been affected by this pandemic. But little by little, they have dealt with the issue and improved the service process. The provision of services to PLWH is less affected and more in the area of harm reduction, for example, the workers in Drop-in centers (DIC) and outreach faced problems providing services to the people who were in the hotspots during the COVID-19 pandemic.” (MD, 42 years old, Male)*.

Said another participant: *“During this period, services such as providing medicines to patients and their treatment process have not been seriously disrupted, and the system has performed well. Since there is a good relationship between the patient and the therapist, the staff do not leave the patient under any circumstances due to their prejudice against the patient. When the indicators were checked, it was shown that among the HIV indicators, most of the diagnosis indicators were affected by the COVID-19 pandemic, and there was no disruption in the rest of the indicators.” (PhD in Epidemiology, 53 years old, Male)*.

**2) Ways of the effect of COVID-19.** Participants believed the COVID-19 pandemic has affected the conditions governing HIV epidemiology and the provision of HIV services in several ways. According to the opinions of the participants in group A, lack of diagnosis or late diagnosis of PLWH, quarantine problems, mental problems, and organizational problems caused by the COVID-19 pandemic, increase risky behaviors among young people and create conflict in families and between couples. In this way, it has caused the creation of sexual behavior outside the framework of marriage and increased the risk of HIV. Among the statements of the participants in this regard, the following can be mentioned:

One of the experts said: *“Before the COVID-19 pandemic, there was entertainment, games, sightseeing, and fun going to cafes or restaurants for young people. All of these were removed due to the restrictions of this pandemic, so they turned to periods accompanied by alcohol and risky behaviors, and other behaviors that increased the risk of HIV transmission. Anyway, instead of this group going after the pastimes that they used to do with their families, they participated in these events, and as a result, it could lead to an increased risk of HIV transmission.” (Infectious Disease Specialist, 40 years old, Female)*.

One of the participants commented on the lack of diagnosis or delayed diagnosis: *“A person with a high-risk behavior has a higher risk of HIV transmission. Perhaps this person was also infected, but during the COVID-19 pandemic, they did not come for testing or came late. Therefore, he might have infected several other people during the time they did not diagnose. If we were not during the COVID-19 pandemic, this person who had high-risk behavior might have come earlier and been diagnosed and treated and would not have infected other people.” (Infectious Disease Specialist, 55 years old, Female)*.

Said another participant: *“As you know, an increase in depression and anxiety following quarantines can lead to an increase in drug and alcohol use. These issues are not specific to the period of the covid-19 pandemic; even years after the pandemic, complications such as depression and anxiety may remain, leading to abuse and increasing the risk of HIV transmission.” (BSc in Psychology, 32 years old, Female)*.

In addition, the pandemic has caused organizational disruptions among service providers and the entire society. Due to the allocation of budgets related to HIV sectors to the COVID-19 sector, financial resources for HIV control services and measures have decreased. In addition, all the media were provided with the news of *COVID-19*, and there were no media to educate society and people at risk about HIV and high-risk behaviors. The following can be mentioned among the participants’ statements: *“Some of our medical centers have two personnel. When one or both of these people got involved in the COVID-19 programs, the counseling centers took on a semi-closed state or were completely closed. In addition, during this period, there was a disruption in the process of granting financial credits to meet the centers’ needs. For example, it has been a year since we have been given any credit. It seems that the COVID-19 program is taking the credit for itself.” (BSc in public health, 30 years old, Female)*.

Another person said: *“Another issue was that all educational media and national media were given access to COVID-19, which means we could not discuss HIV even on World AIDS Day because all sensitivities and concerns, society’s priorities, political views, and health priorities were on the COVID-19 pandemic.” (*Social Studies Specialist, *39 years old, Female)*.

Moreover, participants of group B believed the COVID-19 pandemic has affected their life in several ways, including physical problems, mental and emotional problems, financial problems, changes in the care plan, and impact on high-risk behaviors.

*Physical problems.* During this period, the interviewees were involved in physical issues affecting their health.

One of the PLWH spoke about the immune system’s weakness after contracting COVID-19: *“When I got COVID-19, my immune system was down. I had a severe cough. Even though I was taking medicine, I felt that the medicine did not affect my body. I felt weak and tired. My CD*_*4*_*was at 690; when I got COVID-19, it came to 300.” (Female, 38 years old)*.

*Mental and emotional problems.* One of the challenging issues during the COVID-19 pandemic in this research was mental and emotional problems. The interviewees stated their discomfort due to not participating in gatherings and their fear of contracting COVID-19.

One PLWH said: *“Before the COVID-19 pandemic, I frequently visited my family and friends. When I talked with them, my worries were reduced. But during the COVID-19 pandemic, my communication has become much less, and I feel very lonely and sad.” (Male, 36 years old)*.

*Financial problems.* Several participants talked about the impact of COVID-19 on their financial situation and stated that they suffered much financial pressure due to the loss of their jobs and income.

A woman who used to babysit to support herself and her family explained how she lost her job: *“I was a babysitter. The child’s parents wanted me to stay with them 24 hours a week to ensure I wasn’t exposed to COVID-19, but I couldn’t accept their request because I’m married and have a family of my own. So, I lost my job.” (Female, 32 years old)*.

*Change in the care plan.* Participants stated that many changes occurred in the care process during the COVID-19 pandemic.

One of the participants said about the changes made: *“During this time, in order to protect ourselves, we tried to be less in contact with different people, including those who had the symptoms of COVID-19, and for this reason, we visited the VCT less often, and they gave us more medicine for a few months.” (Male, 52 years old)*.

The COVID-19 pandemic led to a series of changes in providing services, which were not provided in this way before this pandemic. One participant said, *“During the days of COVID-19, I would not leave the house for several months because I was worried about contracting COVID-19. But from the VCT center, they brought the medicines to our door, and this was a perfect situation during the pandemic.” (Male, 25 years old)*.

*Impact on high-risk behaviors.* According to the participants’ statements in this study, the COVID-19 pandemic had different effects on their high-risk behaviors, including; increasing drug injection, decreasing access to condoms, decreasing syringe sharing, and decreasing sexual contact.

One of the people at risk said: *“During the days of COVID-19, the price of drugs increased, and I could no longer get drugs. For the same reason, I used to take it by injection.” (Male, 31 years old)*.

In contrast to the adverse effects of this pandemic, it also had a series of positive impacts on people at risk, which are described here. One of the people at risk said about group drug use: *“I used to use drugs with my friends. But now I am afraid of COVID-19. I try to be alone when taking and not to go with friends.” (Male, 54 years old)*.

Another person at risk commented on the impact of COVID-19 on sexual relations. He said: *I am under financial pressure at this time. I have a small income. I have to spend my income on essential things, and substance use is my priority. If I had sex before, but now it is not a priority for me.” (Female, 38 years old)*.

**3) Healthcare systems reaction.** During the COVID-19 pandemic, the AIDS Office of the Ministry of Health and the Harm Reduction Office of the Welfare Organization took measures to improve the provision of services to PLWH, including providing services through telehealth, dispending HIV drugs for more time and launching campaigns during this period to identify the PLWH. Some of them are mentioned below in the words of experts.

One of the participants said about not stopping the treatment during this period: *“Our patients were those who had not even left their homes for six months, and for them not to stop treatment, we provided the conditions through the helpers and delivered the medicine to them just so that they would not stop treatment.” (BSc, public health, 38 years old, Male)*.

One of the experts said: *“Another measure we made during this period was increasing the intervals between patient visits and giving them more medicine so that their treatment would not be interrupted.” (MD, 40 years old, Male)*.

One of the participants said about launching effective campaigns to identify HIV cases these days: *“In 2021, we launched a campaign called the 20–20 (20 Aban to 20 Azar 2021: equivalent 11 November to 11 December 2021), a campaign in the days close to the World AIDS Day, in this campaign, in addition to informing about the World AIDS Day, several thousand kits were used to detect HIV and obtained favorable results”. (Infectious Disease Specialist, 40 years old, Female)*

**4) Effects on social inequality.** The COVID-19 pandemic has caused an increase in inequality in society.

One of the interviewees spoke about the created inequalities: *“In general, when the systems change from their normal working mode to emergency mode, the quality is lower, and the inequalities increase. Also, vulnerable people are more affected in this era of created inequalities. For example, even though this group is at higher risk, they were not prioritized for the vaccine. Even a series of people did not have identification documents to go and get vaccines.” (PhD in Epidemiology, 48 years old, Male)*.

**5) Opportunities created by the pandemic.** The most critical suggestions presented by the participants in this research were: strengthening the infrastructure to provide the possibility of training and providing remote health services for all people, supplying HIV self-testing services, and training service provider employees to provide services in crisis.

One of the participants said about continuing to provide services online: *“In my opinion, even if this pandemic situation is resolved and ends, we should still have online conditions and learn that it can be beneficial in non-pandemic conditions and can be effective in diagnosis and treatment. This is what I realized during this time.” (MSc, psychology, 35 years old, Female)*.

**6) Suggestions for the future.** Some of the participants in this study believed that the COVID-19 pandemic, along with all the negative points and destructive effects on human health and the provision of services, also it had created opportunities for providing services in the future, including increasing attention to telemedicine, providing opportunities for integration of rapid tests into the HIV surveillance program. It can be used to provide better services in the future.

One of the participants stated about this: *“This is a good opportunity to integrate the discussion of rapid tests in pharmacies; if we use this opportunity, we can easily put rapid tests in pharmacies with minimal sensitivity and encourage people to test themselves and by the way, we can turn COVID-19 pandemic into strength.” (PhD in Epidemiology, 53 years old, Male)*.

## Discussion

The results showed that the provision of HIV services had been affected by the COVID-19 pandemic, these effects were very high at the beginning of the pandemic, and gradually the results of the pandemic have decreased. It can be said that it has returned to normal now. Also, according to the participants’ opinions, diagnostic and preventive services have been more affected, while medical services have been less affected by the COVID-19 pandemic due to the measures taken. According to the results of this study, the COVID-19 pandemic has caused an increase in risky behaviors in society; it has also caused changes in the pattern of drug consumption and sexual behavior. In addition, due to the focus of the COVID-19 pandemic, this pandemic has taken most of the financial credits. Also, this pandemic caused physical problems for some patients. In addition, due to the restrictions created during this period, psychological and economic problems were also among other issues that arose during this period.

According to the results of this study, the COVID-19 pandemic has reduced diagnostic and HIV testing services. The decrease in HIV testing services was the decrease in referrals due to quarantine, people’s fear, and economic problems caused by COVID-19. Part of reducing HIV diagnostic tests has been due to issues related to human resources because the human resources related to HIV services have been employed in the COVID-19 department. A systematic review showed there is an inequality in the provision of HIV services in public and private centers. According to this study, the reduction in the provision of services in public centers can be attributed to several factors, including quarantine restrictions, the lack of staff in the HIV department, as well as the lack of financial resources due to its allocation to the COVID-19 department, which is in line with the results of our study [22]. Considering the extent of the pandemic, it seems that the number of HIV diagnostic tests has decreased in all countries; the study of Rick et al., which was conducted in four countries, showed that HIV diagnostic services have reduced to a large extent in these countries [23]. Also, the results of other studies in Australia and Japan showed a decrease in the number of HIV tests [24, 25]. In Iran, as stated by one of the participants, to compensate for the decline in HIV diagnostic tests, an HIV diagnosis campaign was launched for one month, from 11 November to 11 December 2021. In this campaign, more than 300 new cases of HIV were detected throughout Iran. The number of patients diagnosed in the campaign was far more than those diagnosed in one month in the pre-COVID-19 pandemic. Based on the number of people identified in the HIV diagnosis campaign, the AIDS Department and the provincial HIV experts stated that the number of HIV cases increased during the COVID-19 pandemic. According to the opinions of the participants in this study, due to the closure of recreational facilities and the reduction of recreation among young people, HIV-risky behaviors such as drug and alcohol use and high-risk sexual behaviors have increased among young people and adolescents, so part of the increase in new HIV cases in this era can be due to this reason. Of course, this increase in cases could also be due to the increase in HIV tests during the campaign.

The COVID-19 pandemic has affected the global economy and posed a risk to the economic development of low- and middle-income countries [26]. According to the International Labor Organization (ILO) report, the effects of COVID-19 are so vast that it can make hundreds of millions of people face unemployment and poverty [27]. Due to the economic pressures caused by the sanctions, Iran’s economy has been facing problems [28], and currently, the financial situation has worsened due to the COVID-19 pandemic [29]. The service recipients in this study talked about the economic pressures created in this era and the loss of their jobs and income. Therefore, it is necessary to adopt ways to reduce the financial problems of these people. Some suggestions in this regard include the following: preparing support packages (for example, health and food packages) for this group, as well as delivering these support packages and ART to homes to avoid frequent trips by public transportation and also to reduce fear and worry about getting infected with COVID-19.

According to the findings of the present study, COVID-19 had a positive effect on drug use, especially among those who used drugs in groups but some people prefer to use drugs individually. This change could reduce the risk of needle sharing and HIV transmission. The COVID-19 pandemic had several positive consequences. In this regard, the results of a study in Malaysia showed that people were worried about contracting the COVID-19 infection and the severity of COVID-19 if it was contracted by a smoker [30]. There is a clear association between severe cases of COVID-19 and patients who smoke or have a history of smoking [31, 32]. Fear and anxiety about the COVID-19 virus may make smokers think twice before smoking [30]. In addition, in our study, during the COVID-19 pandemic, people’s sexual relationships were also affected, and these relationships became less outside the framework of marriage. In this regard, studies have shown that after the implementation of travel restrictions around the world, the incidence of sexually transmitted diseases and HIV has shown some reduction [33]. Shortly after the pandemic began, there was an 85% drop in sex among Australian gay and bisexual men. The observed decrease in sexual behavior may be attributed to the increased risk of contracting COVID-19 and the pressure of physical distancing, which can generally decrease libido [34].

To control HIV, patients must be informed about their disease, considering that in Iran, only about 40% of people know about their HIV status; Therefore, paying attention to this matter is required [35]. One of the suggestions made in this context was regarding using self-tests. Some other countries also use self-tests; Brazil’s study indicated these tests were used as telemedicine during the COVID-19 pandemic [36]. In Iran, using self-test kits outside the healthcare system is illegal and not used for self-testing. But considering the stigma toward drug use and high-risk sexual behaviors that lead to people not going to health and treatment centers [37], providing HIV self-test kits outside these centers; can help a lot in increasing the detection of HIV cases.

Among the other effects of the COVID-19 pandemic on HIV-related services in this research was the reduction of mobile service provision in hotspots and gathering centers of people at risk. Among the studies conducted to investigate the effects of the COVID-19 pandemic on the condition of HIV prevention services, most of the studies have examined pre-exposure and post-exposure prophylaxis services, which results showed that in most cases, the preventive medicine services such as pre-exposure and post-exposure-prophylaxis-services-have decreased [38–40]; unfortunately, pre-exposure and post-exposure prophylaxis services are not provided in Iran. A study conducted in New Haven, Connecticut, United States, to investigate the use of harm reduction services among people who inject drugs showed that access to laboratory services, syringes, safe injection equipment, and consultation sessions with doctors during the COVID-19 pandemic had decreased significantly [41]. However, in some studies, it has been shown that with the introduction of the COVID-19 vaccine, the provision of HIV services became better, because the vaccine increased the self-confidence of service providers, and they provided services more completely and comprehensively than before the vaccine came into use [42]. In general, remote medical services and delivery of harm reduction services through couriers and at home are other ways to effectively improve the situation.

Immediate treatment with ART significantly improves the lives of PLWH; therefore, starting treatment on time for patients Field is essential [35]. This study showed that during the COVID-19 pandemic, the initiation of therapy in PLWH was disrupted. Also, the study results showed that COVID-19 indirectly affected people’s living conditions, such as it caused people to lose their jobs and income, affecting their nutritional and shelter, and their mental and psychological conditions. This shows that although the COVID-19 pandemic has had a less direct impact on the HIV treatment status of affected people if the pandemic continues, its indirect effects can lead to treatment disruption. A national study in China showed that during the COVID-19 pandemic, about 35% of PLWH had a higher chance of having an HIV medication disorder, and 2.7% also had a drug use disorder [43]. Also, a study in South Africa showed that the initiation of treatment in new patients in this country was disturbed, but there was no disturbance in treating patients who had been treated [44]. Various strategies have been used to reduce the effects of the COVID-19 pandemic in the continuation of the treatment of PLWH; for example, in China, they have prepared and published a list of centers providing HIV services and drugs. PLWH can go to the nearest center or receive the desired service by mail [12]. Shortly after the pandemic’s start in Iran, new guidelines for treating PLWH were designed and provided to VCT. Also, in some cities such as Mashhad, active matched people were used to deliver pharmaceutical services at the door of homes. In this regard, a study also conducted in California used building relationships with local agencies, especially in the early days of the epidemic, in providing services to patients. According to the researchers of this study, further development of these partnerships may help organizations with limited resources and ensure better coordination of care and services for clients [45]. Therefore, based on our findings, such interventions in Iran also caused the COVID-19 pandemic to have no significant effect on the treatment of patients under treatment.

## Limitations

This study has … limitations. First, some of the interviews were performed on the phone, and FGD was done online, so participants’ body language (The critical aspect of the face-to-face interview) was not considered. Second, the paper presented the short-term effects of COVID-19 on HIV-related services so we suggest that future research consider the long-term effect of COVID-19 on HIV-related services. Despite these limitations, our study offers valuable insights into the impact of the COVID-19 pandemic on providing and receiving HIV services in Iran. One of the advantages of this study is that, because study was done between November 2021 and February 2022, nearly two years after starting the COVID-19 pandemic, study participants experienced different situations regarding the pandemic status, so changes in the experiences and feelings of participants over time have been considered.

## Conclusion

According to the results of this study, many HIV services have been affected by the COVID-19 pandemic, especially at the beginning of the pandemic. However, over time, they have provided services in various ways, such as telemedicine, door-to-door services, and providing referees to patients for a long time to significantly reduce the pandemic’s effects. Considering the level of society’s involvement with the issue of COVID-19 and the surprise caused by this pandemic, many of these effects have been inevitable. However, the participants’ opinions are that we should use this experience and consider the possibility of such emergencies in all policies and design new interventions; this issue will increase the system’s resilience against such troubles. As the world health organization mentioned, improving health systems’ resilience improves preparedness for similar shocks.

## Data Availability

Data will be available upon request submitted to the corresponding author.

## Abbreviations

UNAIDS: United Nations Programme on HIV/AIDS
PLWH: People Living With HIV
ART: Antiretroviral Therapy
VCT: Voluntary Counseling and Testing
NGO: Non-Governmental Organization
FGD: focused group discussions.
